# Caspase-2 Substrates: To Apoptosis, Cell Cycle Control, and Beyond

**DOI:** 10.3389/fcell.2020.610022

**Published:** 2020-12-23

**Authors:** Alexandra N. Brown-Suedel, Lisa Bouchier-Hayes

**Affiliations:** ^1^Hematology-Oncology Section, Department of Pediatrics, Department of Molecular Cell Biology, Baylor College of Medicine, Houston, TX, United States; ^2^William T. Shearer Center for Human Immunobiology, Texas Children’s Hospital, Houston, TX, United States

**Keywords:** caspase-2, PIDD, Raidd, BID, MDM2, apoptosis, cell cycle, tumor suppressor

## Abstract

Caspase-2 belongs to the caspase family of proteins responsible for essential cellular functions including apoptosis and inflammation. Uniquely, caspase-2 has been identified as a tumor suppressor, but how it regulates this function is still unknown. For many years, caspase-2 has been considered an “orphan” caspase because, although it is able to induce apoptosis, there is an abundance of conflicting evidence that questions its necessity for apoptosis. Recent evidence supports that caspase-2 has non-apoptotic functions in the cell cycle and protection from genomic instability. It is unclear how caspase-2 regulates these opposing functions, which has made the mechanism of tumor suppression by caspase-2 difficult to determine. As a protease, caspase-2 likely exerts its functions by proteolytic cleavage of cellular substrates. This review highlights the known substrates of caspase-2 with a special focus on their functional relevance to caspase-2’s role as a tumor suppressor.

## Introduction

Members of the caspase family of proteases are essential for the initiation and execution of apoptosis. When caspases are activated, they cleave a wide variety of substrates that bring about the death of the cell and are responsible for the morphological hallmarks of apoptosis, including membrane blebbing and nuclear condensation. Certain caspase targets have more specific functions that can even regulate non-apoptotic functions of caspases. The classical example of this is the cleavage of pro-IL-1β by caspase-1 to initiate the inflammatory response (Bolivar et al., 2019). Caspase-2 is considered a pro-apoptotic caspase that has a unique role as a tumor suppressor in multiple tissue types. Emerging evidence demonstrates that caspase-2 not only has an apoptotic role, but that it can also contribute to the regulation of other cellular events including cell division. However, there are only a few fully verified caspase-2 substrates that are known and, therefore, how caspase-2 can regulate both apoptosis and non-apoptotic processes to suppress tumors has been difficult to unravel. In this review, we highlight the substrates of caspase-2 and explore possible mechanisms of substrate selection that may regulate the tumor suppressive function of caspase-2.

## The Mechanism of Caspase-2 Activation

**FIGURE 1:**
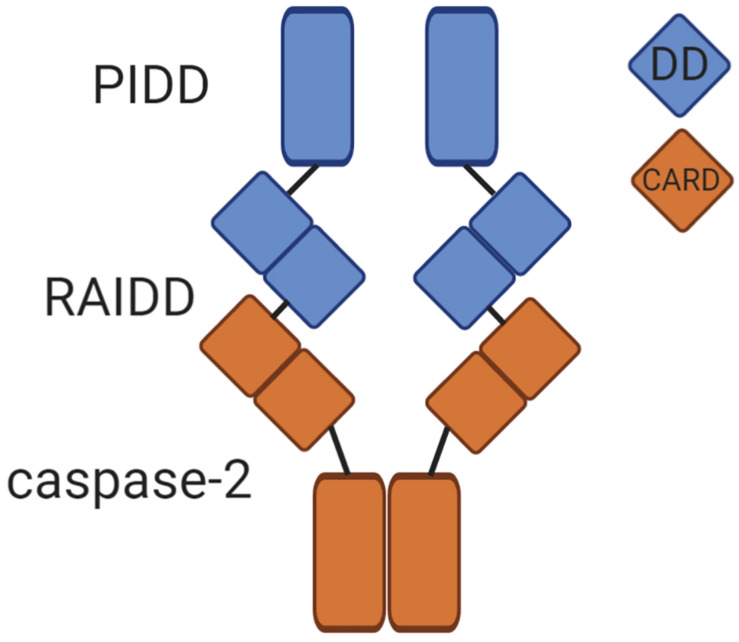
The caspase-2 PIDDosome. The PIDDosome is the best characterized caspase-2 activation platform. PIDD-CC and RAIDD form the scaffold for complex formation via an interaction between their corresponding death domains (DD, blue squares). The CARD of caspase-2 binds the CARD of RAIDD (orange squares), promoting proximity-induced dimerization of caspase-2 monomers. This allows caspase-2 to become fully catalytically active.

Caspase-2 is classified as an initiator caspase, which respond to apoptotic stimuli by initiating the apoptotic cascade. Like other caspases, caspase-2 is comprised of an N-terminal prodomain and a large and small catalytic subunit. A defining characteristic of initiator caspases, the long prodomain of caspase-2 contains a protein interaction motif, specifically a caspase activation recruitment domain (CARD) (Hofmann et al., 1997). This prodomain is essential for initiating caspase-2 activation because it facilitates dimerization of caspase monomers, which is the apical step in initiator caspase activation (Baliga et al., 2004). Dimerization of initiator caspases occurs through induced proximity upon recruitment to high molecular weight complexes called activation platforms. This occurs through protein-protein interactions mediated by conserved domains. For example, caspase-8 is recruited to and is dimerized at the DISC (death inducing signaling complex) by interaction between death effector domains (DEDs) present in caspase-8 and its adaptor protein FADD (Fas-associated protein with death domain). In the APAF-1 (Apoptotic protease activating factor 1) apoptosome, caspase-9 is activated by dimerization mediated by interactions between the CARDs present in APAF-1 and caspase-9 (Boatright and Salvesen, 2003). Caspase-2 is activated in a similar fashion. The activation platform for caspase-2 appears to be the PIDDosome, a molecular complex containing the proteins PIDD1 (p53-inducible protein with a death domain 1) and RAIDD (receptor-interacting protein-associated ICH-1/CED-3 homologous protein with a death domain) (Tinel and Tschopp, 2004; Figure 1). PIDD1 is a p53 response gene that serves as a scaffold for PIDDosome assembly. Upon expression, PIDD1 is constitutively processed by an autocatalytic mechanism into three fragments: PIDD-N, PIDD-C, and PIDD-CC (Tinel et al., 2007). Cleavage of full length PIDD1 to PIDD-N and PIDD-C occurs first, followed by a second cleavage of PIDD-C to yield PIDD-CC. It is the death domain (DD) containing PIDD-CC fragment that is responsible for assembly of the PIDDosome and activation of caspase-2. RAIDD serves as the adaptor molecule to mediate the PIDDosome’s assembly. RAIDD contains both a CARD domain and a DD (death domain) and binds caspase-2 via a CARD-CARD interaction (Duan and Dixit, 1997). Similarly, the DD of RAIDD binds a DD in PIDD1 (Tinel and Tschopp, 2004). A cleavage defective mutant of PIDD1 that could not be processed to PIDD-CC failed to bind RAIDD and did not induce apoptosis after treatment with doxorubicin, demonstrating the necessity of PIDD-CC for PIDDosome assembly and caspase-2-mediated death (Tinel et al., 2007). Assembly of this complex facilitates proximity-induced dimerization, and thus, activation of caspase-2 (Bouchier-Hayes et al., 2009). The crystal structure of the PIDD1 DD/RAIDD DD interaction reveals an asymmetric, oligomeric complex containing five PIDD1 death domains bound to 7 RAIDD death domains (Park et al., 2007). This structure would potentially allow up to seven caspase-2 monomers to be recruited via an interaction with RAIDD. This asymmetry is odd, because only six monomers could dimerize at one time, resulting in three active caspase-2 dimers. The significance of this asymmetry is unknown, but evidence from the asymmetric CARD interactions between initiator caspase-9 and the apoptosome suggests that asymmetric assembly may allow for enhanced stabilization of the complex (Dorstyn et al., 2018).

The primary evidence that caspase-2 belongs to the initiator caspase subgroup is that, similar to caspase-8 and caspase-9, caspase-2 is activated by dimerization and not cleavage (Baliga et al., 2004). *In vitro* studies using recombinant caspase-2 showed that monomeric caspase-2 had no catalytic activity, while a dimeric version of caspase-2 was fully active. When the cleavage site of caspase-2 was mutated to prevent cleavage, the dimeric full length protein retained up to 20% of its catalytic efficiency. This demonstrates that dimerization must occur for activation of caspase-2 (Baliga et al., 2004). After dimerization, cleavage of caspase-2 occurs auto-catalytically. This functions to stabilize the active dimer, resulting in enhanced catalytic efficiency. This is similar to the activation mechanisms for caspase-8 and caspase-9 (Boatright et al., 2003), and distinct to that of the executioner caspases, caspase-3 and caspase-7, which are activated by cleavage (Boatright and Salvesen, 2003). While caspase-2 can be cleaved by caspase-3 (Harvey et al., 1996), unlike executioner caspases, it is not dependent on additional caspases for its proteolytic processing. For example, caspase-2 cleavage induced by PIDD1 overexpression is not impaired in MCF-7 cells lacking caspase-3 (Tinel and Tschopp, 2004). In contrast, in thymocytes deficient in APAF-1 or caspase-9, caspase-2 processing was defective in response to intrinsic apoptosis stimuli like actinomycin D, but loss of caspase-2 did not impair apoptosis (O’Reilly et al., 2002). Together, these studies provide evidence that caspase-2 processing downstream of caspase-3, in the absence of dimerization, is dispensable for cell death in response to stimuli that directly engage the mitochondrial pathway, while caspase-2 cleavage that results from auto-processing following dimerization is more representative of caspase-2 activation.

Despite structural evidence that classifies caspase-2 as an initiator caspase, it possesses unique qualities that set it apart from the other initiator caspases and clouded its initial categorization. The canonical function of initiator caspases is to activate downstream executioner caspases by proteolytic cleavage (Boatright and Salvesen, 2003). Caspase-2 has no detectable enzymatic activity toward other caspases; instead, it cleaves other cellular substrates to promote its functions and indirect activation of downstream caspases (Guo et al., 2002). Early studies of peptide libraries showed that the cleavage specificity of caspase-2 is more similar to executioner caspases than other initiator caspases (Talanian et al., 1997; Thornberry et al., 1997). The most efficiently cleaved caspase-2 substrate peptide, VDVAD, is also efficiently cleaved by executioner caspases (Talanian et al., 1997; Thornberry et al., 1997). A more recent degradomics approach revealed that caspase-2, caspase-3, and caspase-7 were nearly identical in their substrate cleavage motif preference, DEVD, demonstrating the overlap between caspase-2 and executioner caspase substrate specificity (Wejda et al., 2012). This overlap has made it challenging to not only categorize caspase-2, but also to identify its unique physiological substrate pool responsible for executing its functions. Without a full understanding of caspase-2’s targets and their impact on cell death, it has been difficult to correctly place caspase-2 in the apoptotic cascade and to fully understand its physiological functions.

## Caspase-2 Is a Tumor Suppressor

Somewhat unique among the caspase family is caspase-2’s proposed function as a tumor suppressor (Boice and Bouchier-Hayes, 2020). Caspase-2 has been identified as a tumor suppressor in multiple murine models of oncogene-driven cancers. While caspase-2 deficiency alone is not competent to induce tumor formation, its role as a tumor suppressor has been recapitulated by independent groups and in various cancer models. The first demonstration of the tumor suppressor ability of caspase-2 was in an *E*μ*-Myc* model of lymphoma (Ho et al., 2009). *E*μ*-Myc* transgenic mice develop spontaneous B cell lymphomas due to MYC expression from the strong immunoglobulin μ enhancer (Adams et al., 1985). Both partial and complete loss of caspase-2 in combination with the *E*μ*-Myc* transgene accelerated the rate of growth of these tumors (Ho et al., 2009; Manzl et al., 2012). These findings were recapitulated in a second lymphoma model using *Atm*-deficient mice (Puccini et al., 2013). Dual caspase-2/ATM deficiency increased the incidence of tumor formation from 31% in control *Atm*-deficient mice to nearly 64% in *Casp2/Atm* double knockout mice. Further, the average age of tumor onset was decreased in *Casp2/Atm* double knockout mice compared to *Atm* knockout alone. This suggests that loss of caspase-2 not only increased overall tumor incidence but also increased the rate of tumorigenesis. This increased tumorigenesis also resulted in significantly poorer survival of *Casp2/Atm* knockout mice in comparison to loss of *Atm* alone. Caspase-2 also acts as a tumor suppressor in non-hematologic malignancies. Using a model of MMTV/*c-neu* mammary tumor formation, we demonstrated that caspase-2 can also act as a tumor suppressor in the context of an epithelial cancer (Parsons et al., 2013). Deletion of caspase-2 increased tumor incidence and significantly decreased cancer free survival in multiparous female mice. Similarly, loss of caspase-2 has been linked to increased tumor burden in the *Kras*-driven lung cancer model (Terry et al., 2015). In this model, *Kras* lung tumors deficient in caspase-2 were both more numerous and had a higher average volume than tumors induced by Kras alone. This difference in tumor burden has also been observed in a chemically induced model of liver cancer, where caspase-2 deficiency promoted both higher incidence and a significantly increased number of cancerous nodes in the liver after injection with the DNA-alkylating and reactive oxygen species carcinogen Diethylnitrosamine (DEN) (Shalini et al., 2016). However, a more recent article showed results in direct opposition to this where DEN-induced liver tumors were more abundant in wild type mice than in mice deficient in either caspase-2, PIDD1, or RAIDD (Sladky et al., 2020b). The reason for this difference is unclear, as the only notable difference in these models is the use of slightly different mouse strains. Shalini et al. used all male mice on a C57BL/6J background, which has a mutation in the NNT gene associated with glucose-mediated insulin secretion, whereas Sladky et al. used all male C57BL/6N mice, which are wild type for this gene (Freeman et al., 2006). However, the significance of this small difference is uncertain. This is not the only model where caspase-2 has been implicated in tumor progression rather than suppression. In a model of *ThMycn*-induced neuroblastoma, *Th-Mycn/Casp2* null mice had delayed onset of tumor formation, suggesting that caspase-2 potentiated tumor growth in this model (Dorstyn et al., 2014). Finally, loss of caspase-2 had no effect on tumor incidence or growth in irradiation-induced lymphoma or 3-methylcholanthrene-induced fibrosarcoma murine models (Peintner et al., 2015). These distinct effects of caspase-2 on different tumor types strongly suggests that caspase-2 functions in different capacities depending on the cellular context.

The mechanism of tumor suppression by caspase-2 has been difficult to determine. As an established mediator of apoptosis, it may be expected that the caspase-2-deficient tumors described above are more resistant to apoptosis and that this defect in cell death underlies their accelerated tumorigenesis. However, this has been difficult to determine. Caspase-2 was initially identified as a conserved pro-apoptotic cysteine protease. Upon its discovery, it was shown that overexpression of caspase-2 resulted in apoptotic cell death in fibroblasts and neuroblastoma cells (Kumar et al., 1994). It has been implicated in apoptosis induced by a variety of damaging stimuli including heat shock, DNA damage, aneuploidy, and microtubule disruption (Robertson et al., 2002; Tu et al., 2006; Ho et al., 2008; Dawar et al., 2017). However, the importance of caspase-2 for cell death is complicated by observations that loss of caspase-2 is not universally protective from cell death induced by these stimuli in all cell types (reviewed in Bouchier-Hayes and Green, 2012). Indeed, much of the controversy in the caspase-2 field has largely resulted from inconsistencies in the apparent necessity for caspase-2 during apoptosis.

For example, caspase-2-deficient splenocytes are resistant to heat shock-induced death, while Jurkat cells are not (Tu et al., 2006; Shelton et al., 2010). In another example, *E1A/Ras* transformed caspase-2-deficient mouse embryonic fibroblasts (MEF) were found to be resistant to apoptosis induced by irradiation, while their primary counterparts were not (Ho et al., 2009). Further clouding the contribution of caspase-2 to apoptosis, is the comparatively normal phenotype of caspase-2-deficient mice. While knockout of the other initiator caspases results in lethality (Zheng et al., 1999), caspase-2 knockout mice are viable, fertile, and are born at expected Mendelian ratios (Bergeron et al., 1998). These animals have few apoptotic defects, but females do have excess oocytes. With the exception of oocytes, most primary cells from these animals are not particularly resistant to apoptosis. Intriguingly, and in direct contrast with a pro-apoptotic role for caspase-2, facial motor neurons die at an accelerated rate in caspase-2-deficient mice. A second, independently generated, caspase-2-deficient knockout line showed similar phenotypes where primary thymocytes and dorsal root ganglion neurons did not display defects in apoptosis in the absence of caspase-2 (O’Reilly et al., 2002).

Consistent with the unclear apoptotic function for caspase-2, few apoptotic defects are reported in caspase-2-deficient tumor models. In *Atm*-deficient lymphomas, loss of caspase-2 had no effect on cell death, both in terms of background death, and death induced by irradiation as measured by TUNEL staining and gating of sub-G1 DNA content cells, respectively (Puccini et al., 2013). In contrast, sub-cultured primary lymphoma cells from *E*μ*-Myc* lymphomas lacking caspase-2 showed a decreased sensitivity to apoptosis induced by cytoskeletal disruption and irradiation as measured by Annexin V binding (Ho et al., 2009). Whether these results contradict due to the mechanism of action of their respective driver genes (*E*μ*-Myc* vs. *Atm* deficiency) or due to differences in the measurement of cell death (TUNEL staining vs. Annexin V binding) is unclear. It is possible that measurement of a more upstream marker of apoptosis would reveal a more profound apoptotic defect in caspase-2-deficient tumors. Terry et al. (2015) used cleaved caspase-3 as a marker of apoptotic cells in their model of *Kras*-driven lung cancer. Surprisingly, they found that caspase-2-deficient *Kras*-driven lung tumors had a significant increase in apoptotic cells after cisplatin treatment that was not observed in caspase-2 wild-type tumors. This result suggests that caspase-2-deficient tumor cells may be more susceptible to apoptosis and is at odds with the role of caspase-2 as a pro-apoptotic protease. However, this result could be due to other phenomena associated with the loss of caspase-2 as discussed below. In the *Th-Mycn* neuroblastoma model, no difference in cell death was observed, although no treatment was administered (Dorstyn et al., 2014). This could potentially be explained by findings that the neuronal activity of caspase-2 requires RAIDD and not PIDD1 (Ribe et al., 2012). Although this was determined in the context of treatment with damaging stimuli, and proposed that RAIDD was required for caspase-2 mediated death of neurons, it provides a potential mechanism for the disparate effects of caspase-2 in different cell types. The difficulty in uncovering the impact of caspase-2 on apoptosis in a tumor setting is reminiscent of the lack of apoptotic phenotypes in caspase-2-deficient mice (Bergeron et al., 1998). In all tumor models discussed above, tumors were generated on a caspase-2-deficient background. The ability of caspase-2 to suppress tumors by assessing tumorigenesis resulting from acute loss of caspase-2, rather than in fully caspase-2-deficient animals has not yet been assessed. Such an acute model of caspase-2 loss could allow for tissue specific effects of caspase-2 to be revealed. Ultimately, the true contribution of apoptosis to the mechanism of tumor suppression by caspase-2 is still largely unknown. These disparate findings of caspase-2’s varying capacities in inducing apoptosis suggest that, while caspase-2 can induce apoptosis, it may perform other non-apoptotic functions that contribute to tumor suppression.

In the paper by Ho et al. (2009) that first identified caspase-2 as a tumor suppressor, the authors also noted that caspase-2-deficient MEF proliferated significantly faster than their wild-type counterparts, suggesting a defect in cell cycle control in the absence of caspase-2. Investigation of this phenotype revealed that a significantly higher proportion of caspase-2 deficient cells continued to proliferate after irradiation compared to wild-type cells. This suggests that caspase-2 deficient cells failed to arrest after DNA damage. It is generally thought that highly proliferative tumors are subject to replication stress, which can culminate in various forms of DNA damage (Negrini et al., 2010). Whether loss of caspase-2 causes a failure to arrest due to replication stress in tumors is still unknown, but this could be a potential explanation for faster proliferation. This accelerated growth mirrors the more rapid tumorigenesis and increased tumor burden seen in many of the tumor models discussed here. MMTV/*c-neu* mammary tumors that lacked caspase-2 had a higher mitotic index than caspase-2 wild-type tumors (Parsons et al., 2013). In *Atm*-deficient lymphomas, loss of caspase-2 conferred a proliferative advantage without impacting apoptosis (Puccini et al., 2013). This enhanced proliferation was also observed in *Kras*-driven lung tumors deficient in caspase-2 (Terry et al., 2015). However, in non-transformed primary MEF the effect of caspase-2 loss on proliferation was not as dramatic (Manzl et al., 2009). Therefore, similar to the apoptotic phenotypes associated with caspase-2, an oncogenic trigger may be required for this function of caspase-2. Similarly, in the neuroblastoma model, where caspase-2 appeared to act more like an oncogene, defects in proliferation were not observed (Dorstyn et al., 2014). The increased proliferation seen in the absence of caspase-2 in some but not all tumor models suggests that this effect could be tissue or cell type dependent. Identifying the mechanism of this increased proliferation could reveal the mechanism of tumor suppression by caspase-2.

A prevalent phenotype in caspase-2-deficient tumor models results from features associated with increased genomic instability. Genomic instability is a hallmark of cancer (Hanahan and Weinberg, 2000), and is thought to promote tumor heterogeneity and overall tumor survival (Burrell et al., 2013), so this could also be a potential mechanism to explain the tumor suppressor function of caspase-2. MMTV/*c-neu* mammary tumors from caspase-2-deficient animals had a higher proportion of cells with karyomegaly, aneuploidy, and chromosomal aberrations in the form of bizarre mitoses (Parsons et al., 2013). *Atm*-deficient lymphomas display a low level of chromosomal instability, but this was significantly exacerbated in the absence of caspase-2 (Puccini et al., 2013). Incidence of both low and high grade aneuploidy was increased in dual *Casp2*/*Atm*-deficient lymphomas, suggesting that caspase-2 protects against aneuploidy. In support of this function, it was shown that when caspase-2 expression is suppressed in a model of colorectal cancer, aneuploidy increased (Lopez-Garcia et al., 2017). It is possible that prevention of genomic instability is tied to caspase-2’s apoptotic role. In primary splenocytes, it was shown that caspase-2 is required for cell death induced by aneuploidy (Dawar et al., 2017). Aneuploidy was induced by inhibition of PLK1, which is important for centrosome maturation and mitotic spindle formation. *Casp2* knockout cells were more resistant to treatment with a PLK1 inhibitor and became multinucleated. Increased resistance to death and a higher incidence of multinucleation was also observed when caspase-2-deficient cells were treated with the microtubule disruptor Taxol, which also impacts mitotic spindle formation. Primary MEF cells lacking caspase-2 had previously been shown to be more resistant to Taxol, suggesting that caspase-2 is required for death induced by cytoskeletal disruption (Ho et al., 2008). While more recent studies have shown no requirement for caspase-2 in cytoskeletal disruption-induced cell death (Manzl et al., 2009), cytoskeletal disruptors like Taxol and Vincristine are inducers of caspase-2 activation (Bouchier-Hayes et al., 2009; Ando et al., 2017). Thus, aneuploidy resulting from cytoskeletal disruption may be a trigger for caspase-2-mediated apoptosis or may lead to an alternative caspase-2-mediated functional outcome. Therefore, caspase-2 may prevent aneuploidy and genomic instability due to its role in the cell cycle and the increased genomic instability observed in caspase-2-deficient tumors is a result of defective cell cycle regulation as a result of cytokinesis failure.

So, how can an ostensibly pro-apoptotic protein regulate both apoptosis and cell division and how does it have such disparate effects on different tumor models? In the remaining part of this review, we propose that this is achieved through differential targeting of caspase-2 substrates. In short, caspase-2 gains access to different subsets of proteolytic targets in a context-dependent manner, and this access is determined by how caspase-2 is regulated.

## Caspase-2 Substrates

There has been a distinct lack of investigation into the substrates of caspase-2, which has hampered our understanding of how this caspase functions. For example, the MEROPS database of proteases and substrates has cataloged 244 caspase-2 substrates (Rawlings et al., 2018). Caspase-3 has nearly twice the number of recorded substrates in the MEROPS database. However, the more telling comparison lies elsewhere. The over 400 caspase-3 substrates listed in the MEROPS database have been compiled from 222 individual studies. In comparison, the 244 caspase-2 substrates come from a mere 13 studies. Of these, 95% come from a single study, performed by Julien et al. (2016). While this landmark study identified the majority of known caspase-2 substrates, it highlights the lack of attention caspase-2 substrates have received in the field.

To date, the largest-scale characterization of caspase-2 substrates was performed using N-terminal degradomics (Julien et al., 2016). Not only were many unique caspase-2 substrates identified, but by performing quantitative enzymology on the top hits from the degradomics screen, the potential physiological relevance of these substrates was addressed. BID, MDM2, and Golgin-160, the three known substrates for caspase-2, were not identified in this screen, highlighting the limitations of this type of *in vitro* approach for identifying caspase substrates. In addition, the relevance and veracity of the majority of the substrates that were identified have not yet been validated in physiological settings. Due to these problems, there are only a few verified substrates for caspase-2 that have been identified and characterized *in vivo*. Because of this, it is not our intention to provide an extensive list of every substrate that has been published as a putative caspase-2 substrate. Rather, we provide a discussion of the more well-studied caspase-2 substrates, with attention paid to how these substrates may be executing the functions of caspase-2 that could contribute to tumor suppression (Table 1). We contend that there exists a number of additional, as yet unidentified, caspase-2 substrates, the access to which provides critical decision points for the execution of caspase-2 function.

**TABLE 1:** Caspase-2 substrates.

Substrate	Caspase-2 cleavage motif (human)	Position (human)	Conserved in mouse (Y/N)	Mouse cleavage motif	Position (mouse)	Cleaved by other caspases?
MDM2	DVPD	362	Yes	DVPD	359	Caspase-3
Golgin-160	ESPD	59	Yes	GSPD	59	No
Golgin-160	SEVD	311	Yes	SEAD	308	Caspase-3 and caspase-7
Bid	LQTD	60	Yes	LQTD	59	Caspase-8 and caspase-3
Ku80	GDVD	726	Yes	GDVD	725	Unknown
ICAD	?	?	?	?	?	Caspase-3
Rictor	?	?	?	?	?	Unknown
eIF4B	DRKD	563	Yes	DRKD	563	Caspase-3*
HDAC4	DVTD	289	Yes	DVTD	288	Caspase-3 and caspase-7
Tau	KPVD	314	Yes	KEQD	289	Unknown
HTT	NGKD	213	Yes	NGKD	212	Caspase-3
S1P	LYGD	846	Yes	LYGD	846	Unknown

*With a much lower efficiency than caspase-2.

### BID—The Primary Mediator of Caspase-2 Induced Apoptosis

Early demonstrations of caspase-2 as an apoptotic protease also yielded the first clue that caspase-2 functions in the intrinsic apoptotic pathway. Apoptosis induced by overexpression of caspase-2 was blocked by expression of BCL-2, which prevents mitochondrial outer membrane permeabilization (MOMP) (Kumar et al., 1995). The primary way caspase-2 initiates apoptosis through the mitochondrial pathway is by proteolytic cleavage of the BH3-only domain protein BID (Guo et al., 2002; Bonzon et al., 2006). Caspase-mediated cleavage of BID results in its conversion to tBID, which promotes MOMP, cytochrome c release, executioner caspase activation, and apoptosis (Luo et al., 1998). Accordingly, *Bid*-deficient MEF were resistant to cell death induced by caspase-2 overexpression, and heat shock (Bonzon et al., 2006). In addition, it has been shown that heat shock induces caspase-2 dimerization prior to MOMP (Bouchier-Hayes et al., 2009). Inhibition of caspase-2-induced cell death by BCL-2 and cleavage of BID places caspase-2 upstream of the mitochondria.

Although BID is one of the few caspase-2 substrates with a clear function, BID is not exclusively cleaved by caspase-2 (Luo et al., 1998; Slee et al., 2000). Caspase-8 can also cleave BID, with up to four times greater efficiency than caspase-2 (Bonzon et al., 2006). Although this higher cleavage efficiency would seem to suggest a redundant function for caspase-2 in BID cleavage, caspase-8 is an initiator caspase that is activated by extrinsic rather than intrinsic stimuli (Varfolomeev et al., 1998). Cleavage of BID by caspase-8 represents a point of convergence between the intrinsic and extrinsic apoptotic cascades (Li et al., 1998). One report suggests that in certain cell types, caspase-2 may participate in extrinsic apoptosis induced by TRAIL upstream of BID cleavage (Wagner et al., 2004). This study showed that in HCT116 and T3M4 cells, knockdown of caspase-2 significantly reduced TRAIL induced apoptosis, though not to as great an extent as knockdown of caspase-8 or BID. This suggests that caspase-2 could have a redundant role to caspase-8 in the TRAIL pathway. However, these effects were not recapitulated in Hela cells, suggesting that this is not a general mechanism of caspase-2 activation (Wagner et al., 2004).

**FIGURE 2:**
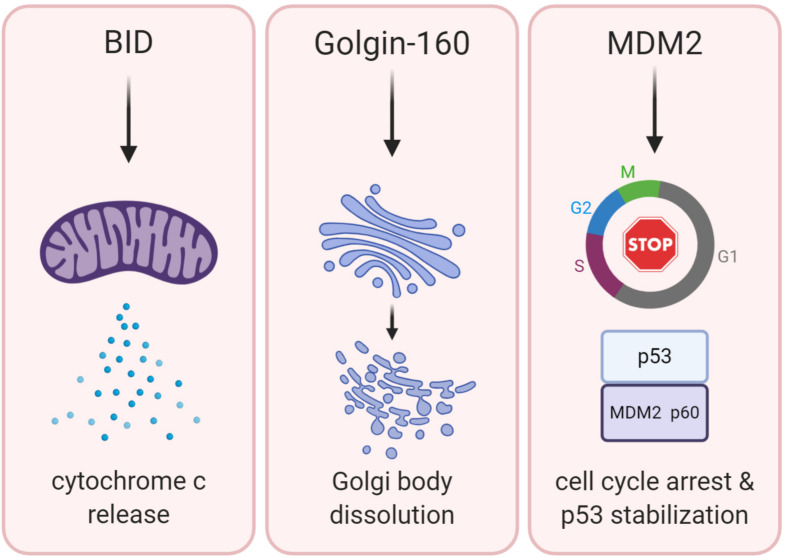
BID, Golgin-160, and MDM2 are established caspase-2 substrates. The potential downstream functions of caspase-2-mediated cleavage of these substrates are shown. Cleavage of BID (*left panel*) promotes permeabilization of the outer mitochondrial membrane, releasing cytochrome c and activating downstream executioner caspases inducing apoptosis. Cleavage of Golgin-160 (*center panel*) induces Golgi fragmentation during apoptosis. MDM2 cleavage by caspase-2 (*right panel*) leads to the stabilization of p53 and promotion of cell cycle arrest.

Because cleavage of BID is a well-established contributor to apoptosis, BID proteolysis could be a potential mechanism to explain tumor suppression by caspase-2. Therefore, BID would induce MOMP to induce apoptosis, which would promote removal of cancerous cells (Figure 2). Although there are few clear examples of an apoptotic defect in caspase-2 deficient tumor models, BID cleavage has not been examined in these models. Determining if BID cleavage is defective in these models would be valuable in determining the true mechanism of tumor suppression by caspase-2 as it would suggest that apoptosis is impaired in these tumors, even though it may be masked *in vivo*. However, there is limited information as to whether BID phenocopies the tumor suppressive properties of caspase-2. One study showed that *Bid*-deficient mice develop a myeloproliferative disorder that resembles chronic myelomonocytic leukemia (CMML) as it progresses (Zinkel et al., 2003). Further, this study showed that *Bid*-deficient cells were resistant to the extrinsic death-inducing ligands, anti-Fas and TNF-α. Their resistance to intrinsic apoptotic damage was not investigated, so the contribution of caspase-2 is difficult to extrapolate. However, metaphase spreads from BID-deficient tumors displayed advanced chromosomal aberrations, including trisomy and translocation. These chromosomal abnormalities are similar to the genomic instability observed in caspase-2-deficient tumors, but it is unclear if caspase-2 is involved in BID-deficient tumorigenesis or how generalized this phenomenon is.

While cleavage of BID appears to be the main route for caspase-2-induced apoptosis, evidence of caspase-2-dependent apoptosis that does not require BID exists. For example, in *Tp53* null cells, caspase-2 has been linked to apoptosis independent of both mitochondrial permeabilization and downstream activation of caspase-3. This cell death only occurred when the cell cycle checkpoint protein Chk1 was inhibited, and was not inhibited by overexpression of BCL2 (Sidi et al., 2008). In another example, purified caspase-2 was shown to trigger cytochrome-c release from isolated mitochondria without the addition of cytosolic factors (Robertson et al., 2004). Caspase-8, which can also cleave BID, was not able to replicate this effect, suggesting that cytochrome c release did not occur simply due to cytosolic contamination. Therefore, additional caspase-2 substrates may exist that induce apoptosis independent of MOMP or BID.

### MDM2—A Key Link to the p53 Pathway

MDM2 is a caspase-2 substrate that has the potential to reveal the mechanism of tumor suppression by caspase-2 due to its own ability to regulate both apoptosis and cell cycle (Figure 2). Best known as the E3 ubiquitin ligase responsible for targeting p53 to the proteasome for degradation, MDM2 is cleaved by caspase-2 at Asparagine 367 (Oliver et al., 2011). MDM2 is a key negative regulator of p53 levels and stability (Horn and Vousden, 2007). Furthermore, MDM2 is a p53 target gene, comprising a negative feedback loop that mediates levels of both p53 and MDM2 itself (Momand et al., 1992). Cleavage of MDM2 by caspase-2 removes the critical RING finger domain, which is responsible for the ubiquitination of p53 by MDM2 (Fang et al., 2000). Oliver et al. (2011) showed that both PIDD1 and RAIDD expression generates a p60 MDM2 fragment that is abolished by mutation of the Asp367 cleavage site. They further showed that while both caspase-2 and caspase-3 can directly cleave MDM2 *in vitro*, caspase-3 was not activated by their experimental conditions, suggesting that, in their study, MDM2 is primarily cleaved by caspase-2. Caspase-2 also cleaved MDM2 in response to DNA damage induced by doxorubicin, showing that MDM2 is a physiologically relevant substrate. Because cleavage of MDM2 removes the RING domain, this results in reduced degradation of p53, increasing both the levels and activity of p53. Furthermore, immunoprecipitation experiments have shown that the MDM2 p60 fragment binds p53, and even outcompetes full-length MDM2 for p53 binding. Binding of MDM2 p60 to p53 stabilizes rather than degrades p53. Therefore, cleavage of MDM2 by caspase-2 appears to be important for the maintenance of p53 levels after DNA damage. In support of this, a recent study demonstrated that activation of caspase-2 after DNA damage promotes sustained levels of p53 rather than oscillatory protein dynamics (Tsabar et al., 2020). This sustained p53 response prevented continual cell cycling in the presence of DNA damage. Importantly, this was reported to be dependent on caspase-2-mediated cleavage of MDM2, allowing p53 levels to rise. Again, this shows that caspase-2 has an important role in protection from aberrant cell cycling, which could be critical for its ability to suppress tumor formation.

The discovery of MDM2 as a caspase-2 substrate revealed an important connection to the critical p53 tumor suppressor pathway. The tumor suppressing functions of p53 are linked to its ability to trigger both apoptosis and cell cycle arrest (Chen, 2016), key processes that may also underlie tumor suppression by caspase-2. However, current evidence suggests that the caspase-2/MDM2/p53 axis may serve to promote cell cycle arrest, rather than apoptosis. It has been reported that caspase-2-mediated MDM2 cleavage leads to p53 accumulation and p21-dependent cell cycle arrest in human lung cancer cells (Fava et al., 2017). This was observed under conditions of “mitotic catastrophe,” a variety of conditions including cytokinesis failure, increased mitotic duration, and microtubule disruption. Inhibitors generating these phenotypes resulted in activation of caspase-2 as monitored by the appearance of cleaved MDM2, which was reportedly produced in a caspase-2 specific manner. Although these conditions resulted in robust cleavage of MDM2, this occurred in the absence of notable cell death. Cytokinesis failure resulted in increased polyploidy, which was less severe in the presence of caspase-2, suggesting that caspase-2 limits the extent of this phenotype. This is consistent with observations that caspase-2-deficient tumors display enhanced aneuploidy and mitotic aberrations (Parsons et al., 2013; Lopez-Garcia et al., 2017). Caspase-2 has also been implicated in the constraint of polyploidy in the liver during organogenesis and regeneration (Sladky et al., 2020a). Cytokinesis failure in human lung cancer cells led to MDM2 cleavage, p53 accumulation, and p21-mediated cell cycle arrest (Fava et al., 2017). Caspase-2 deficiency completely blocked MDM2 cleavage, and resulted in a failure to accumulate p53 and arrest after inhibition of cytokinesis. It is proposed that this failure to arrest is what leads to the enhanced polyploidy seen in caspase-2-deficient lung cancer cells. This suggests that caspase-2 mediates cell cycle arrest – and limits polyploidy—by acting through p53. However, there was no observed defect in p53 accumulation when caspase-2-deficient cells were exposed to DNA damage or increased mitotic duration triggered by nocodazole (Fava et al., 2017). Therefore, caspase-2-mediated stabilization of p53 leading to cell cycle arrest as a mechanism to limit polyploidy may be specific to stimuli resulting in cytokinesis failure. This may also be a cell-type specific phenomenon, as it was reported that caspase-2-deficient U2OS cells still effectively induce p53 and cell cycle arrest in response to the same inhibitors used by Lim et al. (2018).

Ultimately, it is unlikely that cleavage of MDM2 represents the sole mechanism of tumor suppression by caspase-2. While caspase-2-deficient cells failed to induce p53 in response to cytokinesis failure, it was clear that caspase-2 was not required for p53 induction under other conditions (Fava et al., 2017). Furthermore, while *Tp53*-deficient animals develop severe spontaneous tumors, dual loss of caspase-2 does not exacerbate this phenotype (Manzl et al., 2013), suggesting that these tumor suppressors may function independently of each other. However, based on the evidence that suggests caspase-2 is necessary for cell cycle arrest following cytokinesis failure, it is possible that a failure to cleave MDM2 may be at least partially responsible for the observable ploidy defects in caspase-2-deficient tumors (Parsons et al., 2013; Puccini et al., 2013).

While MDM2 represents a direct link between caspase-2 and p53, other reports point to a connection between caspase-2 and p53 independent of MDM2. Terry et al. (2015) found that in a cohort of lung cancer patients, caspase-2 mRNA levels were significantly lower in patients with wild type p53 than in patients with a mutation in p53. This suggests that p53 may act to suppress caspase-2, and is in agreement with an earlier report that showed lower caspase-2 transcript levels in the presence of p53 in H1299 tumor cells (Baptiste-Okoh et al., 2008). This report also showed lower protein levels of caspase-2 in the presence of p53, but since cells were treated with daunorubicin to induce p53, this may be due to processing of full length caspase-2, which was not shown. Caspase-2 has also been suggested to be required for the expression of *p21*, a key p53 target gene responsible for mediating cell cycle arrest (Sohn et al., 2011). Interestingly, this was reported to be independent of the catalytic activity of caspase-2, which suggests a potential non-catalytic function for caspase-2. However, the tumor suppression of caspase-2 has been shown to be dependent on caspase-2’s catalytic activity (Ren et al., 2012), so it is unlikely that this contributes to tumor suppression in the absence of an additional enzymatic function of caspase-2.

Caspase-2 has been implicated in an alternative “Chk1 suppressed” apoptotic pathway in p53 null cells (Sidi et al., 2008). Chk1 is a key G2/M cell cycle checkpoint, responsible for arresting cells with DNA damage before the completion of cell division (Zhang and Hunter, 2014). It was shown that when Chk1 is inhibited, caspase-2 restores apoptosis in both zebrafish and human p53 null cells induced by irradiation (Sidi et al., 2008). However, Chk1 inhibition failed to significantly restore DNA damage-induced cell death in cells from various *Tp53* null mouse tissues (Manzl et al., 2013). Moreover, the cell death that did occur was shown to be independent of caspase-2 in *Casp2*/*Tp53* double knockout cells. MDM2 cleavage was not investigated in these p53-deficient models, so it is unclear if caspase-2 targets MDM2 as a substrate in these conditions. Furthermore, although MDM2 is primarily recognized for its degradation of p53, it does not exclusively act on p53 (Riley and Lozano, 2012). In the absence of p53, cleavage of MDM2 could still have an effect by failing to degrade other targets, although this has not yet been investigated.

### Golgin-160—A Surprising Mediator of Cell Fate?

Golgin-160 is a member of a superfamily of structural Golgi body proteins. While Golgins are generally thought to be important for maintenance of Golgi structure, siRNA studies for Golgin-160 suggest that it is dispensable for the structural integrity of this organelle (Hicks et al., 2006). Rather, it has been suggested that it may be important for correct transport and shuttling of certain cellular proteins like the beta-1 Adrenergic Receptor (Hicks et al., 2006; Gilbert et al., 2014). Golgin-160 was identified as a caspase-2 substrate following observations that showed endogenous caspase-2 co-localized with Golgi body markers (Mancini et al., 2000). Upon investigation, it was revealed that caspase-2 cleaves Golgin-160 in two locations. The first of these, Asparagine 59, is exclusive to caspase-2, while the second cleavage site, Asp 311, can also be cleaved by caspases -3 and -7. Caspase-generated fragments of Golgin-160 were observed after induction of apoptosis with staurosporine, suggesting that Golgin-160 may be an apoptotic substrate. It was also shown that the caspase-2 specific fragment of Golgin-160 (D59 cleavage) appeared at an earlier time point than other caspase generated Golgin fragments. This is consistent with the role of caspase-2 as an initiator caspase, which would be activated prior to executioner caspases. A D59 mutant of Golgin-160 that could not be cleaved by caspase-2 significantly delayed the normal fragmentation of the Golgi body that occurs during apoptosis. This suggests that caspase-2 participates in the dissolution of the Golgi body during apoptosis, supporting a role for caspase-2 in the apoptotic cascade (Figure 2). However, this is incongruous with the findings that Golgin-160 is dispensable for maintenance of Golgi body architecture (Hicks et al., 2006), suggesting a potential other outcome of this cleavage.

Interestingly, multiple studies have reported finding caspase-generated fragments of Golgi proteins in the nucleus (Hicks and Machamer, 2002; Sbodio et al., 2006; Mukherjee and Shields, 2009). Indeed, the N-terminal domain of Golgin-160 contains a cryptic nuclear localization signal that is hidden in the full length protein (Hicks and Machamer, 2002). Mutants of Golgin-160 representing various caspase cleavage products demonstrate that multiple Golgin-160 fragments localize readily to the nucleus. Of these, two fragments have the potential to be generated exclusively by caspase-2, and one has the potential to be cooperatively produced by caspase-2 and caspase-3 (Mancini et al., 2000). A study in Hela cells showed that this cooperatively produced fragment could be prevented from localizing to the nucleus by expression of another Golgi protein, GCP60, which bound to the Golgin-160 fragment, preventing its translocation (Sbodio et al., 2006). Preventing this translocation actually rendered the cells significantly more sensitive to staurosporine, suggesting that nuclear translocation of Golgin-160 fragments may actually exert a pro-survival effect. While this was only determined for a specific Golgin-160 fragment in which caspase-2 only plays a supporting role (and is not strictly required, as the fragment could also be produced by caspase-3 alone or caspase-3 in tandem with caspase-7), it is tempting to speculate that caspase-2 and Golgin-160 could be part of a cell fate-determining signaling pathway. There is a precedent for this seemingly unusual signaling mechanism. Another Golgi protein, p115, can also be cleaved by caspases after apoptotic stimuli, yielding fragments that localize to the nucleus (Mukherjee and Shields, 2009). In the case of p115, the nuclear fragments were actually found to potentiate apoptosis, suggesting that there is a complex network of signaling between the Golgi body and nucleus that regulates cell fate. While it is unknown how these Golgi body protein fragments impact cell fate from the nucleus, it has been speculated that this could be due to a change in transcriptional programs (Mukherjee and Shields, 2009). While this is certainly a possibility, it would be important to determine if this a specific, targeted transcriptional reprogramming, or a general non-specific interference with existing transcription. In addition, it will also be important to determine which protein fragments promote either death or survival, and which caspases are responsible for generating these fragments.

### Other Substrates Point to Alternative Functions for Caspase-2

In addition to the substrates discussed above, there are several other caspase-2 substrates proposed in the literature (Table 1). While cleavage of many of these by caspase-2 has only been shown *in vitro* or in overexpression-based experiments, some are worth discussing here based on their potential functional relevance. For example, two proposed substrates, Ku80 and ICAD, could link caspase-2 to the DNA damage response, which could underlie the observations of increased genomic instability in caspase-2-deficient tumors. Ku80 is a key member of the complex responsible for mediating non-homologous end joining (NHEJ). It interacts with another member of the complex, DNA-PK_cs_, to repair DNA breaks that occur throughout the cell cycle (Gottlieb and Jackson, 1993). It has been reported that caspase-2 promotes repair of double-stranded DNA breaks through non-homologous end joining (NHEJ) by cleaving Ku80 (Yan et al., 2017). The last 12 residues of the C-terminal end of Ku80 are reported to be important for this interaction with DNA-PK_cs_ (Gell and Jackson, 1999). Caspase-2 cleaves Ku80 at D726 at the extreme C-terminus and the removal of these few amino acids was reported to increase the affinity of the interaction between Ku80 and DNA-PK_cs_ (Yan et al., 2017). Importantly, this enhanced interaction increased the ability of cells to repair etoposide-induced DNA damage. This finding provides a potential explanation for the genomic instability observed in caspase-2-deficient tumors. However, many of these experiments were done using a C-terminally tagged version of Ku80, so further investigation is needed to determine if the tag impacted the cleavage of Ku80 and to see if this occurs physiologically and promotes the tumor suppressor effect of caspase-2. ICAD (Inhibitor of caspase-activated DNase) is an inhibitor of one of the key mediators of apoptosis, CAD, and is inactivated by caspase-mediated cleavage (Sakahira et al., 1998). When ICAD is unable to bind CAD, CAD degrades DNA, resulting in the characteristic nuclear condensation at the terminal stages of apoptosis (Sakahira et al., 1998). Lower levels of CAD activation resulting from minimal caspase activity have been shown to induce DNA damage and promote genomic instability (Ichim et al., 2015). It has been shown that caspase-2 can cleave ICAD *in vitro*, albeit with much less efficiency than caspase-3 (Dahal et al., 2007). Therefore, caspase-2 could promote DNA damage or even apoptosis through ICAD cleavage. However, the only study to demonstrate caspase-2-dependent ICAD cleavage used a Tat-caspase-2 fusion protein that had a higher cleavage efficiency toward ICAD than recombinant caspase-2 (Dahal et al., 2007) and this observation has yet to be confirmed in cells by endogenous caspase-2.

Substrates that contribute to caspase-2’s role in cancer may do so through more indirect mechanisms by regulating transcription or translation. Two examples of these are translation initiation factor eIF4B, and transcriptional corepressor histone deacetylase 4 (HDAC4) (Paroni et al., 2004; Wejda et al., 2012). HDAC4 cleavage at D289 by caspase-2 produces an N-terminal fragment that when expressed leads to transcriptional repression and also appears to induce apoptosis (Paroni et al., 2004). eIF4B has been shown to have a caspase-2 specific cleavage site at D563, but the functional impact of this cleavage on translation has not been assessed (Wejda et al., 2012). The global changes in transcription and translation caused by cleavage of these substrates is unknown, but could represent a potential mechanism to impact cell division or other physiological processes in which caspase-2 has been implicated, like autophagy, metabolism, or differentiation, that may contribute to its regulation of tumor growth.

Additional putative caspase-2 substrates may be linked to other potential functions of caspase-2 that may not have an obvious cancer relevance. For example, caspase-2 has been linked to neurodegeneration (Hermel et al., 2004; Zhao et al., 2016). Caspase-2 reportedly cleaves Huntingtin (HTT), a neuroprotective protein of the central nervous system (Hermel et al., 2004). Cleavage of HTT generates a cytotoxic protein fragment thought to contribute to the neuronal cell death characteristic of Huntington’s disease. Expression of a catalytically inactive caspase-2 inhibited cleavage of HTT and reduced cell death, suggesting that caspase-2 may participate in cell death associated with neurodegeneration caused by Huntington’s disease. Caspase-2 has also been reported to cleave tau, a fibril forming protein thought to contribute to Alzheimer’s pathology (Zhao et al., 2016). In a mouse model, cleavage of tau by caspase-2 had a detrimental effect on cognitive function, again pointing to a link between caspase-2 and neurodegenerative diseases. Cleavage of Rictor (rapamycin-insensitive companion of mTOR) by caspase-2 has been implicated in the inhibition of an mTOR/Akt signaling cascade to promote the normal progression of synaptic pruning (Xu et al., 2019). In humans, impaired synaptic pruning has been linked to mental disorders such as autism and schizophrenia, and indeed, loss of caspase-2 is associated with incomplete synaptic pruning and dysfunctional behavioral traits in mice (Xu et al., 2019). However, in these studies, cleavage of Rictor was only observed after overexpression of caspase-2 and the cleavage site has not been identified. This does not necessarily mean that Rictor is not a physiologically relevant substrate, as the authors note that endogenous levels of Rictor are low, so overexpression of caspase-2 may have increased the detection threshold of its cleavage products. Degradation of Rictor by caspase-2 had a similar effect on synaptic pruning as knockdown of Rictor, suggesting that cleavage by caspase-2 renders the protein inactive. Interestingly, Rictor amplification has been noted in a number of different cancer types such as small cell lung cancer, colorectal cancer and esophageal squamous cell carcinoma (Zhao et al., 2020), indicating that cleavage of this putative substrate may play a role outside of neuronal processes to impact cancer progression.

Caspase-2 has also been implicated in obesity, metabolism, and the development of non-alcoholic fatty liver disease (Machado et al., 2016). Interestingly, caspase-2-deficient mice fed a high-fat diet were protected from the development of obesity and associated phenotypes like diabetes and hepatic steatosis (Machado et al., 2016). Further, caspase-2 is required for the progression from non-alcoholic fatty liver disease to non-alcoholic steatohepatitis, which is considered a risk factor for further liver damage that promotes hepatocellular carcinoma (Kim et al., 2018). One of the mechanisms that has been explored to explain this is elevated endoplasmic reticulum (ER) stress (Kim et al., 2018). ER stress has been found to upregulate caspase-2 expression, which in turn activated sterol regulatory element-binding proteins (SREBP) leading to the buildup of cholesterol and triglycerides. Notably, an earlier report suggests that caspase-2 is under transcriptional control of SREBP2, which could form a positive feedback loop promoting further SREBP activation (Logette et al., 2005). This activation of SREBP was reportedly due to caspase-2-dependent cleavage of site 1 protease (S1P), which once cleaved, becomes active and competent to activate SREBP. Given the relationship between non-alcoholic fatty liver disease and liver cancer, it is interesting to speculate that via this S1P/SREBP mechanism, caspase-2 could ultimately be potentiating the risk of liver cancer. However, this has not yet been investigated, and there is other evidence suggesting that caspase-2 may have a more specialized role in liver development as discussed below (Sladky et al., 2020a).

Like the substrates discussed in more detail above, a primary problem in making definitive conclusions about the function of substrates cleaved by caspase-2 is the lack of evidence suggesting that these substrate cleavage sites are exclusive to caspase-2. For example, while enzymology suggests that eIF4B is cleaved most efficiently by caspase-2, it can still be cleaved by caspase-3 and -7 *in vitro* (Wejda et al., 2012). HDAC4 on the other hand, appears to be much more efficiently cleaved by caspase-3 *in vitro*. Neither of these scenarios is enough to determine whether a substrate is exclusive to caspase-2 (or not*) in vivo*. Indeed, while there is enough evidence to suggest that the substrates of caspase-2 play critical roles in apoptosis, cell cycle control, and beyond, the key question remains: how does caspase-2 select these substrates to moderate different functions?

## Does Substrate Selection by Caspase-2 Direct Its Function?

**FIGURE 3:**
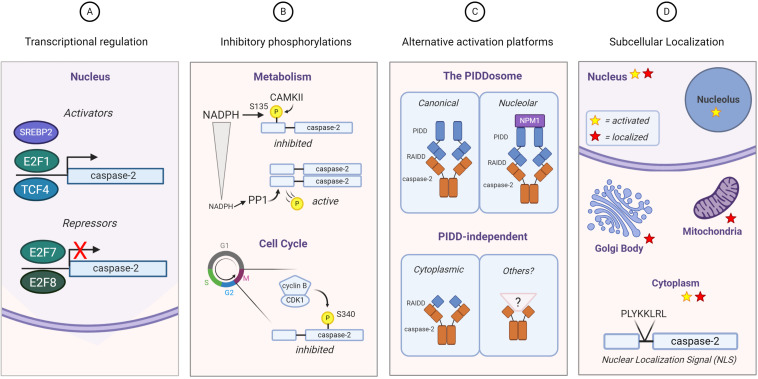
Regulation of caspase-2 expression, activity, and localization. Substrate selection by caspase-2 could be directed by **(A)** transcriptional regulation: E2F family transcription factors both activate (E2F1) and repress (E2F7 and E2F8) caspase-2 transcription. TCF4 and SREBP2 activate caspase-2 transcription; **(B)** phosphorylation: Caspase-2 is phosphorylated on multiple residues. The known phosphorylation events of caspase-2 result in its inhibition, which can be regulated by metabolism (*top*) and the cell cycle (*bottom*); **(C)** differential activation platform assembly: The canonical PIDDosome contains both PIDD1 and RAIDD. Caspase-2 can be activated by complexes lacking one or both of these elements. NPM1 is required for the nucleolar activation complex; **(D)** subcellular localization: Caspase-2 contains a classical nuclear localization signal and it has been found outside the nucleus associated with the Golgi body and mitochondria. Activation of caspase-2 has also been observed specifically in the nucleolus induced by DNA damage, as well as more generally throughout cytoplasm induced by heat shock and microtubule disruption. Red stars represent sites where caspase-2 is expressed and yellow stars represent sites where caspase-2 is activated.

Given the insufficiency of studies on caspase-2 substrates, it is not surprising that there is even less literature that directly examines the regulation of caspase-2 substrate selection. However, the existing body of work on caspase-2 does present several possible mechanisms that could be harnessed to regulate substrate selection (Figure 3).

### Regulation of Caspase-2 Gene and Protein Expression

The first of these potential mechanisms is temporal regulation of caspase-2 activity via either transcriptional or translational mechanisms (Figure 3A). While caspase-2 is generally thought to be a ubiquitously expressed protein, there is some evidence that suggests it may be regulated in these ways. As previously discussed, data from lung cancer patients suggested that caspase-2 mRNA levels were lower in patients with mutated p53, suggesting a level of transcriptional control (Terry et al., 2015). However, whether this is directly controlled by p53 is unknown. A recent report examining caspase-2 in liver development and regeneration places caspase-2 and its activating component PIDD1 under transcriptional control of E2F family members E2F1, E2F7, and E2F8 (Sladky et al., 2020a). E2F1 induced robust expression of both PIDD1 and caspase-2, while E2F7 and E2F8 resulted in transcriptional repression (Sladky et al., 2020a). In support of these findings, caspase-2 transcript levels were higher in E2F7/E2F8 null mouse liver extracts. Furthermore, E2F1 also upregulates E2F7 and E2F8, producing a negative feedback loop to control caspase-2 and PIDD1 expression. It is important to note that not only does this mechanism control expression of caspase-2, but also its ability to be activated by its canonical activating platform by regulating PIDD1 transcription. Ultimately, this loop was reported to activate caspase-2 to constrain polyploidization in the liver that occurs during development (Sladky et al., 2020a). While this phenomenon has not yet been studied in the tumor models where caspase-2 has been shown to play a role, E2F1 is known to promote tumorigenic properties like proliferation, drug resistance, and metastasis (Putzer and Engelmann, 2013). In a study of colorectal cancer patients, Lopez-Garcia et al. (2017) reported that mutation of the transcriptional activator BCL9L was associated with increased aneuploidy. Using a colorectal cancer cell line, this group demonstrated that BCL9L represses caspase-2 at the transcriptional level, resulting in lower protein levels. This transcriptional repression was linked to the inhibition of transcription factor TCF4, which promotes caspase-2 expression by binding near the transcriptional start site of caspase-2. This repression of caspase-2 resulted in increased aneuploidy in colorectal cancer cell lines, thought to exacerbate genetic variability and drug resistance in tumors. Interestingly, BCL9L mutations were highly correlated with p53 mutations, which have also been reported to reduce caspase-2 expression levels (Terry et al., 2015). Therefore, it is possible that this is a cooperative repression between p53 and BCL9L.

### Post-translational Regulation of Caspase-2

Caspase-2 can also be regulated by post-translational modification (Figure 3B). In *Xenopus* oocytes, it was found that nutrient stores regulate cell death via caspase-2 (Nutt et al., 2005). Excess NADPH resulted in an inhibitory phosphorylation event on caspase-2 at S135, while deprivation of NADPH resulted in reduced phosphorylation at this site and enhanced caspase-2 activity and cell death. This phosphorylation was mediated by calcium/calmodulin-dependent protein kinase II (CaMKII). This finding was recapitulated in mouse oocytes, suggesting an important, conserved regulatory mechanism (Nutt et al., 2009). This site can be dephosphorylated by a direct interaction with protein phosphatase-1 (PP1) and can be protected from dephosphorylation by protein 14-3-3zeta, which binds phosphorylated caspase-2, thus preventing interaction with PP1. Phosphorylation of caspase-2 at a different site, S340, has been linked to cell cycle control. It has been observed that the morphological changes seen during mitosis are notably similar to those that occur during apoptosis, like nuclear membrane dissolution. Andersen et al. (2009) showed that caspase-2 is spontaneously processed to its mature form in interphase cell extracts. However, in mitotic cell extracts, this processing is inhibited, preventing caspase-2 mediated cell death during mitosis. Furthermore, they showed that this inhibition is dependent on caspase-2 being phosphorylated on S340. Because caspase-2 phosphorylation on S135 is regulated by metabolism of NADPH (Nutt et al., 2005), nutrient availability was examined in interphase and mitotic extracts, but there was no difference (Andersen et al., 2009). Rather, caspase-2 phosphorylation on S340 was regulated by the mitotic cycle itself. The mitosis promoting kinase cdk1-cyclinB1 was responsible for phosphorylating caspase-2 at S340, resulting in its inhibition. Interestingly, this site was vulnerable to dephosphorylation by PP1, but the action of PP1 appeared to be repressed during mitosis. This regulatory mechanism seems to be important for the demonstrated role of caspase-2 in mitotic catastrophe (Vitale et al., 2017). Mutation of the S340 phospho-site resulted in significantly enhanced cell death in cells arrested in mitosis compared to wild-type cells (Andersen et al., 2009). This finding suggests the existence of an important caspase-2-mediated safeguard against improper cell division. Because S340 is vulnerable to PP1 dephosphorylation, but PP1 is suppressed during mitosis, it may be that extended mitotic duration (symptomatic of mitotic damage) allows PP1 to be re-activated, allowing caspase-2 to be activated. This would promote apoptosis of a cell that is unable to sufficiently repair any mitotic damage, suggesting that caspase-2 could be a “mitotic timer.”

Recently, another phosphorylation event has been demonstrated to be important for successful progression through mitosis. This site, S384, is highly conserved and is phosphorylated by Aurora B kinase, a critical mitotic kinase that controls cytokinesis (Lim et al., 2020). Phosphorylation at this site was induced by PLK1 inhibition, resulting in mitotic catastrophe as described earlier. Interestingly, phosphorylation at S384 prevented cleavage of both MDM2 and BID, suggesting that this modification prevents caspase-2 activation. Interestingly, a phospho-mimetic mutant of this site was still able to be recruited to its activation platform, as measured by a fluorescent readout of caspase-2 induced proximity dimerization. It did, however, reduce auto-processing of caspase-2, which could explain the abrogation of MDM2 and BID cleavage. This study also performed structural analysis that suggests that S384 phosphorylation impacts the conformation of the substrate binding pocket of caspase-2, providing a potential explanation for the reduced auto-cleavage and MDM2/Bid cleavage. Importantly, this study ultimately showed that a S348 phospho-mimetic mutant of caspase-2 resulted in increased ploidy and resistance to cell death after mitotic stress. In contrast to this, another group recently showed that mutation of the S384 phosphorylation site to alanine to prevent phosphorylation blocks caspase-2 processing after DNA damage with cisplatin. This suggests that, in the context of DNA damage, phosphorylation at S384 is actually necessary for caspase-2 processing. However, this group was not able to identify phosphorylation at this site under these conditions, and importantly, a phospho-mimetic mutant did not restore the processing of caspase-2. It is possible that this site is differently regulated during mitosis and DNA damage, which could account for these conflicting findings. However, this more recent study did corroborate the idea that S384 is critical for substrate binding; not by forming a bond with the substrate itself, but by stabilizing Arg-378, which is responsible for binding to the critical Aspartate reside of the target substrate. These studies highlight the importance of caspase-2 phosphorylation at the same residue in different cellular contexts, and demonstrate that post-transcriptional modifications can impact substrate cleavage.

Regulation of caspase-2 expression and activation by post-translational modifications could provide a level of substrate specificity by modulating the amount of available active caspase-2. Thus, substrates that are cleaved most efficiently by caspase-2 would be targeted at the lowest doses of caspase-2, while others would not be cleaved unless caspase-2 was maximally active. However, few studies have explored if there is a dosage dependent effect of caspase-2 on different substrates. In the latter study exploring S384 phosphorylation, both BID and MDM2 cleavage was completely abrogated by S384 phosphorylation suggesting, at least in this case, that it is a binary on-off effect on caspase-2 activation rather than a dynamic response.

### Assembly of Distinct Activation Platforms

Caspase-2 may be regulated toward cleaving different substrate targets through the assembly of distinct caspase-2 activation platforms (Figure 3C). While the PIDDosome is considered the canonical activation platform for caspase-2, its necessity for caspase-2 activation and subsequent physiological outcomes such as cell death has been debated in the literature (Bouchier-Hayes and Green, 2012). For example, primary thymocytes from PIDD1-deficient mice did not display reduced susceptibility to apoptosis induced by a variety of damaging stimuli including DNA damage (Manzl et al., 2009). Further, loss of PIDD1, RAIDD, or caspase-2 had no effect on cytochrome c release in SV40 immortalized fibroblasts induced by etoposide (Manzl et al., 2009), which is at odds with the established role for caspase-2 as an upstream mediator of cytochrome c release in response to DNA damage (Robertson et al., 2002). Caspase-2 was also found to elute in high-molecular weight complexes in the absence of both PIDD1 and RAIDD (Manzl et al., 2009), suggesting that it may have other activating complexes that are still unknown. Tumor suppression by caspase-2 has also been reported to be independent of both PIDD1 and RAIDD in certain models. In a model of c-*Myc*-induced lymphomagenesis, loss of caspase-2 accelerated tumor growth, but loss of PIDD1 suppressed tumor formation, suggesting that caspase-2 does not depend on PIDD1 to suppress tumors (Manzl et al., 2012). The observation that loss of PIDD1 suppressed tumor formation may be due to PIDD1’s known role in activating the pro-survival NFκB pathway in response to genotoxic stress (Janssens et al., 2005). Similarly, loss of RAIDD does not phenocopy the effects of loss of caspase-2 on tumor suppression since RAIDD deficiency had no impact on Eμ-Myc-induced lymphomagenesis (Peintner et al., 2015).

Other potential activators of caspase-2 have been reported. For example, caspase-2 has been proposed to be a component of the TNFR complex, the TRAIL receptor complex, and the CD95 complex (Duan and Dixit, 1997; Droin et al., 2001; Wagner et al., 2004; Lavrik et al., 2006). However, the requirement for caspase-2 for TNF, TRAIL, or CD95-induced apoptosis is unclear. Although antisense-mediated downregulation of caspase-2 has been shown to decrease CD95-induced apoptosis in U937 cells (Droin et al., 2001), caspase-2 failed to facilitate CD95-induced apoptosis in the absence of caspase-8 in Jurkat cells (Lavrik et al., 2006). A more recent study suggests that caspase-2 augments CD95-associated STAT1 activation, suggesting a potential alternate function for caspase-2 at the DISC (Qadir et al., 2020). In the TRAIL pathway, siRNA-mediated silencing of caspase-2 was shown to reduce TRAIL-induced apoptosis in certain cell types (Wagner et al., 2004). A subsequent report suggested that caspase-2 can prime cancer cells for TRAIL-induced death and that caspase-2 is required for TRAIL-induced caspase-8 cleavage (Shin et al., 2005). Although the authors suggested that caspase-2 directly cleaved caspase-8, they did not provide evidence for this and other reports have demonstrated that caspase-2 has no catalytic activity toward other caspase family members (de Craen et al., 1999; Guo et al., 2002). In addition, the latter two TRAIL studies relied heavily on an siRNA oligo that was later shown to have off-target effects (Lassus et al., 2002). Therefore, the exact function, if any, that caspase-2 plays in the TRAIL complex is unclear.

Additional components of caspase-2 activation platforms have been described. TRAF2 has been shown to bind to caspase-2 in a complex that appears to be devoid of PIDD1 (Robeson et al., 2018). In contrast, BubR1 has been proposed as a negative regulator of PIDDosome formation by competing with RAIDD for binding to PIDD1 (Thompson et al., 2015). It is possible that the assembly of different caspase-2 activation platforms or the presence or absence of regulatory molecules in the PIDDosome could result in a caspase-2 enzyme that has variable activity and therefore has variable targeting efficiency for different substrates. Interestingly, there are non-canonical functions of caspase-2 where the activation platform remains unknown. One such report places caspase-2 at the heart of an ER-stress driven inflammatory response (Bronner et al., 2015). In this mechanism, ER stress triggers activation of NLRP3, which translocates to the mitochondria and activates caspase-2 to promote the release of cytochrome c and mitochondrial DNA, resulting in activation of caspase-1, an inflammatory caspase (Bronner et al., 2015). Whether this results in either apoptosis or another form of cell death is not clear, but given the release of mitochondrial factors, it is certainly possible that caspase-2 promotes cell death in this mechanism. This is thought to occur independently of NLRP3’s canonical role as a key member of the NLRP3 inflammasome signaling platform (Bronner et al., 2015). Because NLRP3 functions as part of an activation platform for caspase-1, it would be interesting to determine if NLRP3 participates directly or indirectly in the activation of caspase-2 at the mitochondria. Notably, it is thought that ER stress is a common feature across many types of cancers. Although the pro- vs. anti-tumor impacts of ER stress are still debated in the field (reviewed in Oakes, 2020), it is possible that caspase-2 has been overlooked as a contributor to cell death resulting from this phenotype.

### Regulation of Caspase-2 Localization

Caspase-2 expression has been localized to the nucleus, cytoplasm, mitochondria, and Golgi body (Mancini et al., 2000; Paroni et al., 2002; Lopez-Cruzan et al., 2016; Figure 3D). Caspase-2 contains a classical nuclear localization signal (NLS), located in the C-terminus of its prodomain (Baliga et al., 2003). Mutation of a critical, conserved lysine residue in this NLS abolishes the nuclear localization of caspase-2 but how localization of caspase-2 impacts activation and downstream functions is still unclear. Although caspase-2 has this NLS, and has been reported to induce apoptosis from the nucleus (Paroni et al., 2002), stimuli such as heat shock and cytoskeletal disruption have been shown to activate caspase-2 in the cytoplasm and not in the nucleus (Bouchier-Hayes et al., 2009). To resolve this confusion, we investigated the localization of caspase-2 activation following DNA damage and reported that caspase-2 is activated by the PIDDosome in the nucleolus, while its activation in the cytoplasm is PIDD1-independent but RAIDD-dependent (Ando et al., 2017). This activation is highly stimulus specific, where DNA damaging agents are most efficient at activating caspase-2 in the nucleolus, while other stimuli such as cytoskeletal disrupters only activated caspase-2 in the cytoplasm or nucleus. We found that nucleolar caspase-2 activation is dependent on inclusion of nucleophosmin (NPM1) in the PIDDosome complex by interacting with PIDD1 itself. Surprisingly, in PIDD1 or NPM1-deficient cells, overall levels of caspase-2 activation were not impacted, but activation of caspase-2 specifically within the nucleolar compartment was significantly reduced. This finding suggests that PIDD1 is dispensable for general caspase-2 activation, in agreement with other published work (Manzl et al., 2009), but is required for DNA damage-induced activation specifically within the nucleolus. Consistent with the identification of a nucleolar PIDDosome, PIDD-CC, the fragment responsible for activating caspase-2, has been shown to localize to the nucleus and cytoplasm in unstimulated cells but was concentrated in the nucleolus after UV-irradiation (Pick et al., 2006). This shift could represent either translocation or degradation of PIDD1 following an activating stimulus.

This bifurcation between caspase-2 activation in the cytoplasm and nucleolus could allow access to different substrates in each location. However, the possibility of a nucleolar substrate pool for caspase-2 has not been investigated. Together with the stimuli specific localization patterns of caspase-2 activation, distinct activation platforms in different locations in the cell suggests that the localization of caspase-2 activation is a regulated process and this may be critically important for its downstream functions. The ability of caspase-2 or upstream PIDDosome components to translocate between different cellular compartments is a potential mechanism of caspase-2 regulation. For example, while NPM1 is a predominantly nucleolar protein, it has many roles that necessitate shuttling throughout the cell. Notably, it can localize to the centrosome, where it is thought to suppress centrosome duplication (Wang et al., 2005; Liu et al., 2007). This could also allow NPM1 to play a role in the activation of caspase-2 via the PIDDosome in response to supernumerary centrosomes as reported by Fava et al. (2017), but this has not been investigated. The primary localization of caspase-2 expression, as opposed to activation, has been difficult to determine. GFP-tagged caspase-2 localizes primarily to the nucleus, but does not appreciably translocate out of the nucleus until late in the apoptotic process, presumably due to compromised nuclear integrity (Paroni et al., 2002). One report in HeLa cells suggested that caspase-2 can translocate out of the nucleus in response to hydrogen peroxide and etoposide, but the activation status of this translocated caspase-2 was not determined (Tinnikov and Samuels, 2013). It was shown that this translocation occurred despite treatment with Leptomycin B, a well-established Exportin I inhibitor but was abrogated by another inhibitor, N-Ethylmaleimide (NEM), which alkylates cysteine thiol groups in proteins. How and why NEM specifically prevents caspase-2 translocation was not determined. In direct contrast to this, findings from immortalized MEF suggest that caspase-2 translocated into the nucleus after DNA damage induced by irradiation (Manzl et al., 2009). A more recent structural analysis suggested that the protein 14-3-3, masks the nuclear localization signal of caspase-2 when bound (Smidova et al., 2018). This would suggest that 14-3-3 binding would promote caspase-2 localization in the cytoplasm, but this was not tested in cells. These mechanisms of potentially restricting the activation of caspase-2 to a specific localization in the cell highlights the importance of substrate access. Even if a substrate is readily cleaved by caspase-2, if the localization of either of these is restricted, it introduces a level of regulation determined by the localization of this protease.

## Closing Remarks

The investigations into caspase-2 substrates to date have failed to provide evidence that a single substrate is responsible for all the functions of caspase-2. However, in contrast to the many caspase-3 substrates that have been identified, it is likely that the limited number of caspase-2 substrates is indicative of a more directed function of this caspase. So rather than “death by a thousand cuts” for caspase-3 (Martin and Green, 1995), it may be a case of “death by 20 cuts” for caspase-2. Or, given the non-apoptotic roles of caspase-2, we could possibly paraphrase this to “life by 20 cuts.” While catalytic independent functions of caspase-2 have been described, such as in the regulation of p21 expression (Sohn et al., 2011), tumor suppression associated with caspase-2 can be blocked by mutation of its catalytic cysteine (Ren et al., 2012). Therefore, the most likely route to tumor suppression is through proteolytic cleavage of substrates. It is only through the identification and investigation of additional caspase-2 substrates that we will reveal the cellular processes in which caspase-2 is actively involved, to clarify the important role of this caspase as a safeguard against cancer.

## Author Contributions

ANB-S wrote the manuscript and generated the figures. LB-H wrote and edited the manuscript. Both authors contributed to the article and approved the submitted version.

## Conflict of Interest

The authors declare that the research was conducted in the absence of any commercial or financial relationships that could be construed as a potential conflict of interest.
